# Applications of Surface Plasmon Resonance for Advanced Studies Involving Nucleic Acids

**DOI:** 10.59566/ISRNN.2024.0101044

**Published:** 2024-12

**Authors:** Katelynn Pranger, Kenya Rosas, Dmitriy Khon, Emil F. Khisamutdinov

**Affiliations:** 1 Department of Chemistry, Ball State University, Muncie, IN 47306, USA;; 2 Department of Chemistry and Biochemistry, St. Mary’s University, San Antonio, TX 78228, USA

**Keywords:** Nucleic Acid Nanoparticles, Dissociation Constant, Bioconjugation, Biophysics

## Abstract

Surface plasmon resonance (SPR) is increasingly recognized as one of the most widely used techniques for studying nucleic acid interactions. The main advantage of SPR is its ability to measure the binding affinities and association/dissociation kinetics of complexes in real-time, in a label-free environment, and using relatively small quantities of materials. The method is based on the immobilization of one of the binding partners, ligand, on a dedicated sensor surface. Immobilization is followed by the injection of the other partner, analyte, over the surface containing the ligand. The binding is monitored by subsequent changes in the refractive index of the medium close to the sensor surface upon injection of the analyte. In the field of Nucleic Acid, SPR has been intensively used in the study of various artificial and naturally occurring RNA/DNA molecules interaction with large molecular weight mass proteins and small organic molecules because of its ability to detect highly dynamic complexes that are difficult to investigate using other techniques. This mini review aims to provide a short guideline for setting up SPR experiments to identify nucleic acid complexes and assess their binding affinity or kinetics. It covers protocols for (i) nucleic acid immobilization methods, including biotin-streptavidin, metal ion-based affinity, and amine coupling, (ii) analyte-binding analysis, (iii) affinity and kinetic measurements, and (iv) data interpretation. Determining the affinity and kinetics of nucleic acid interactions through SPR is essential for gaining insights into molecular-level binding mechanisms, thus supporting advancements in nucleic acid nanotechnology. The review also highlights the various sections of SPR applications in nucleic acid research, including nucleic acid-probe immobilization, interactions with biomolecules, aptamer studies, and small molecule binding, concluding with perspectives on future developments in the field.

## INTRODUCTION

For over a decade, nucleic acid engineering has been gaining significant momentum, as pointed out in the first meeting of the International Society of RNA Nanotechnology and Nanomedicine^1^. This extensive research effort is driven by the tremendous potential RNA holds for both basic research and clinical applications^2–5^. RNA, a natural biopolymer, plays a critical role in numerous essential life mechanisms. Understanding RNA’s folding, structure, and functions enables the rational design of RNA-based therapies and nanodevices for biomedical use. As the field has progressed, several RNA therapies have been approved for clinical use, including two notable SARS-CoV-2 mRNA vaccines rapidly developed during the COVID-19 pandemic^6–8^. Despite these advancements, numerous challenges in translating RNA therapeutics into broader biomedical applications persist^9, 10^. However, with an increasing number of research teams dedicated to overcoming these barriers, the emerging RNA field continues to advance toward curative goals.

To fully harness the therapeutic potential of RNA and other nucleic acid-based technologies, precise methods for studying their interactions, stability, and functionality are essential. In this regard, innovative analytical techniques have emerged as invaluable tools, helping to bridge the gap between laboratory research and clinical applications. Among these, Surface Plasmon Resonance (SPR) has gained prominence due to its ability to provide real-time, label-free analysis of biomolecular interactions, which is especially advantageous in nucleic acid research. As research into RNA and other nucleic acids advances, the role of SPR in decoding these interactions grows, offering unprecedented insights into nucleic acid-based diagnostics and therapeutics^11^.

SPR operates as a sophisticated label-free optical biosensing technology, leveraging the excitation of surface plasmons in a metal-dielectric interface to detect minute variations in the refractive index attributable to biomolecular interactions at the sensor surface^12^. This technique is characterized by its high sensitivity, exceptional reliability, precise selectivity, and reproducibility. SPR is extensively employed for the real-time kinetic analysis of biomolecular interactions and the quantitative detection of various biological and chemical analytes, whether in labeled or unlabeled states. Recent advancements have demonstrated the utility of SPR biosensors in the detection and analysis of nucleic acids, highlighting their potential in nucleic acid research and diagnostics^13–16^.

Leveraging an array of signal amplification strategies, such as nanoparticle enhancement, super-sandwich assembly, streptavidin/biotin complexation, antibody amplification, enzymatic reactions, triple helix formation, and catalytic hairpin assembly (CHA), the intrinsic limitations of SPR in detecting low-concentration biomolecules can be effectively addressed^17, 18^. This renders SPR highly suitable for clinical diagnostics. In particular, advancements in nucleic acid nanotechnology have significantly bolstered the performance and sensitivity of SPR-based assays. RNA and DNA nanoparticles, characterized by their exceptional biocompatibility, extensive specific surface area, diverse structural configurations, and robust biological mimicry, have emerged as pivotal in enhancing SPR biosensing capabilities^1, 19–21^. These nanoparticles facilitate numerous biosensing functions and applications, reinforcing their role in the development of highly sensitive and specific diagnostic tools for clinical and research applications.

This minireview aims to elucidate recent advancements in nucleic acid affinity studies employing Surface Plasmon Resonance technology, emphasizing its utility in probing interactions with various ligands. The review outlines the theoretical framework and foundational principles of SPR, followed by a detailed exposition of the different SPR modalities and their respective capabilities. Subsequently, it provides a comprehensive summary of contemporary applications of SPR in the investigation of both artificial nucleic acids, such as aptamers and DNA/RNA nanoparticles, and naturally occurring nucleic acids, highlighting their interactions with a diverse array of ligands. The review seeks to underscore the pivotal role of SPR in advancing our understanding of nucleic acid-ligand interactions and its potential in enhancing the sensitivity and specificity of nucleic acid-based biosensing and diagnostic methodologies.

## SPR WORKING PRINCIPLE

**Fig. 1.**
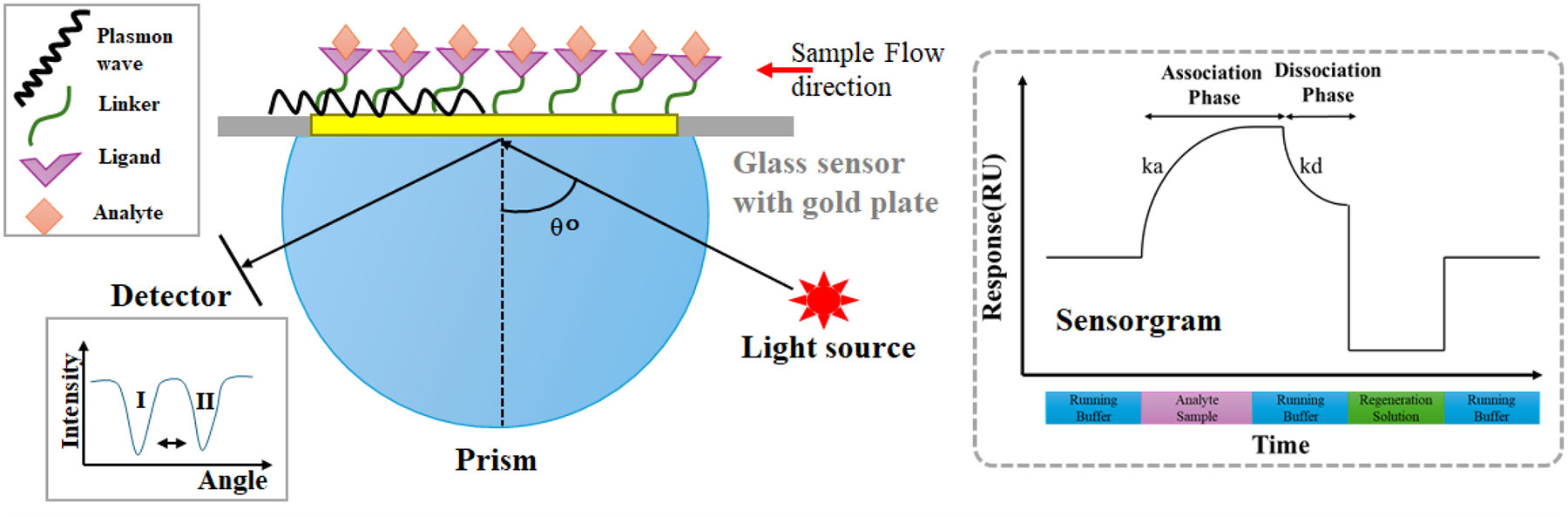
Working Principle of Surface Plasmon Resonance for Biomolecular Interaction Analysis. Polarized light is directed at a metal-dielectric interface (e.g., a gold sensor chip surface). When light is incident at a specific angle (θ°), it excites surface plasmons, generating a resonance effect that decreases the intensity of reflected light. The resonance angle shifts in response to refractive index changes at the sensor surface, which occur when an analyte binds to an immobilized ligand. The resulting sensorgram (inset) graphically represents the interaction in real time, with the response signal (y-axis) indicating the binding event and dissociation phase as the analyte binds to and dissociates from the ligand on the sensor surface.

SPR relies on the excitation of surface plasmons (collective oscillations of electrons) at the interface between a thin metal layer (usually gold) and a dielectric medium (typically the sample solution) (Fig. 1). When polarized light hits this metal-dielectric interface at a specific angle, it causes resonance, resulting in a decrease in reflected light intensity^22, 23^. This angle shift is highly sensitive to changes in the refractive index at the surface, allowing SPR to detect even minute biomolecular interactions, such as ligand-analyte binding^24, 25^.

SPR enables the quantitative measurement of interaction kinetics, including association (binding) and dissociation rates, as well as affinity constants (${\text{K}}_{\text{D}}$). Typically, interactions are described using a 1:1 binding model, where a ligand (B) immobilized on a sensor surface interacts with an analyte (A) in solution to form a complex (AB). This interaction is mathematically represented by the association and dissociation reactions as:

$$A+B\overset{k_{a}}{\to }AB\;\text{and}\;AB\overset{k_{d}}{\to }A+B$$

where $k_{a}$ represents the association rate while $k_{d}$ represents dissociation rate. Here, the equilibrium dissociation constant, ${\text{K}}_{\text{D}}$, which is an indicator of binding affinity, is given by the ratio of the dissociation and association rate constants:

$$K_{D}=\frac{k_{d}}{k_{a}}$$

In an SPR experiment, the ligand is immobilized onto the sensor surface, and the analyte flows over it, binding to form a complex on the sensor surface. The observed signal, ${\text{R}}_{\text{t}}$, is directly proportional to the concentration of AB complexes formed at the surface, which in turn depends on ligand density. For a single ligand, the rate of complex formation is:

$$\frac{dR_{t}}{dt}=k_{a}[\text{A}]\left(R_{max}-R_{t}\right)-k_{d}R_{t}$$

Where [A] is the analyte concentration in the solution, $R_{\text{max}}$ is the capacity of immobilized ligand on the surface in millidegrees, and $\left(R_{max}-R_{t}\right)$ is equal to the number of binding sites that are unoccupied at a certain time. The $R_{t}$ value can be used without converting into absolute ligand concentration in the equation if it is rearranged:

$$\frac{dR_{t}}{dt}=k_{a}[\text{A}]R_{max}-\left(k_{a}[\text{A}]+k_{d}\right)R_{t}$$

The time-dependent response RtR_tRt is then given by

$$R_{t}=\frac{k_{a}\left[\text{A}\right]R_{max}}{k_{a}\left[\text{A}\right]+k_{d}}\left(1-e^{-\left(k_{a}\left[\text{A}\right]+k_{d}\right)t}\right)$$

For the dissociation phase, where only the AB complex is present, the dissociation rate is proportional to the complex concentration:

$$\frac{-\text{d}[\text{AB}]}{\text{d}t}=k_{d}[\text{AB}]\;\text{or}\;k_{d}=-\frac{1}{R_{t}}\frac{dR_{t}}{dt}$$

Through these equations, both the association $\left({\text{k}}_{\text{a}}\right)$ and dissociation $\left({\text{k}}_{\text{d}}\right)$ rate constants can be determined experimentally, yielding insight into the affinity (KD) of the ligand-analyte interaction.

Finally, for experimental determination relevant to $R_{\text{max}}$, the maximum binding signal, the following equations are applied:

$$R_{max}=\left(R_{i}-R_{\text{blank}}\right)\times \text{S}\times \frac{{\text{MW}}_{\text{A}}}{{\text{MW}}_{\text{L}}}$$

$$R_{max}=R_{im}\times \text{S}\times \frac{{\text{MW}}_{\text{A}}}{{\text{MW}}_{\text{L}}}$$

Where ${\text{MW}}_{\text{A}}$ is a mass of analyte A and ${\text{MW}}_{\text{L}}$ is a mass of a ligand L.

## OPTIMIZING SPR EXPERIMENTS: DATA QUALITY, SENSOR SELECTION, AND EXPERIMENTAL CONDITIONS

Ensuring high-quality SPR data requires attention to sensorgram characteristics, model selection, sensor optimization, and experimental conditions. Sensorgrams should exhibit curvature rather than linear profiles, as most biomolecular interactions fit a simple 1:1 binding model. In general, fitting data to complex models should be avoided unless there is substantial evidence of multi-state or cooperative binding^26–28^.

Experimental optimization begins with sensor selection, SPR sensors are typically composed of a glass substrate with a gold nanoparticle layer and a functional chemical coating to immobilize ligands^29–32^. Common sensor types include covalent coupling, capture coupling, and hydrophobic capture sensors^33^. Covalent coupling sensors, such as those with carboxyl and amine groups or plain gold surfaces, allow for targeted ligand immobilization based on chemical compatibility. Capture coupling sensors, such as biotin-streptavidin and NTA for His-tagged ligands, and hydrophobic capture sensors, like liposome capture, are tailored to specific ligand types and binding mechanisms. The right sensor type is crucial for minimizing non-specific binding (NSB), which can introduce false-positive signals. To control NSB, preliminary binding tests with a bare sensor may be conducted. If NSB is present, optimizing conditions (e.g., buffer pH, blocking agents like BSA, non-ionic surfactants, and ionic strength) can help reduce unintended analyte interactions.

Sample preparation is equally critical, as buffers must be appropriately pH-adjusted, degassed, and filtered to prevent interference from refractive index mismatches. Using freshly prepared buffers, particularly when additives are unstable, helps maintain sample integrity. Consistency between sample and buffer composition minimizes background noise. Mass transfer effects can also impact data quality^34^. When diffusion to the sensor surface is slower than the binding interaction, mass transfer limitations may occur, particularly in fast-binding reactions, resulting in linear rather than exponential sensorgrams^35^. To mitigate mass transfer effects, higher flow rates can be applied, or ligand density on the sensor can be adjusted to reduce diffusion limitations. Injection of analytes at multiple flow rates can reveal mass transfer impacts, while fitting models that incorporate mass transport parameters can account for these effects mathematically^36^.

To accurately determine binding constants, varying analyte concentrations should be used to generate multiple binding curves^36, 37^. A preliminary binding test can establish the lowest effective analyte concentration. Analyte concentration is then increased in a standard stepwise fashion (typically threefold increments) to ensure well-spaced sensorgrams for kinetic analysis^38^. Finally, effective regeneration of the sensor surface between injections is essential, particularly for analyte-ligand complexes with low dissociation rates. Commonly used regeneration buffers—such as acids for proteins and antibodies, NaOH for nucleic acids, IPA for lipids, SDS for peptide and protein interactions, and high-salt solutions for ionic interactions—facilitate the removal of bound analyte, allowing the sensor to be reused and ensuring accurate binding kinetics for successive injections. The choice of regeneration buffer depends on the stability and affinity of the ligand-analyte complex, with stronger agents needed for slower-dissociating complexes^39,40^.

## NUCLEIC ACID PROBES AND IMMOBILIZATION STRATEGIES

**Fig. 2.**
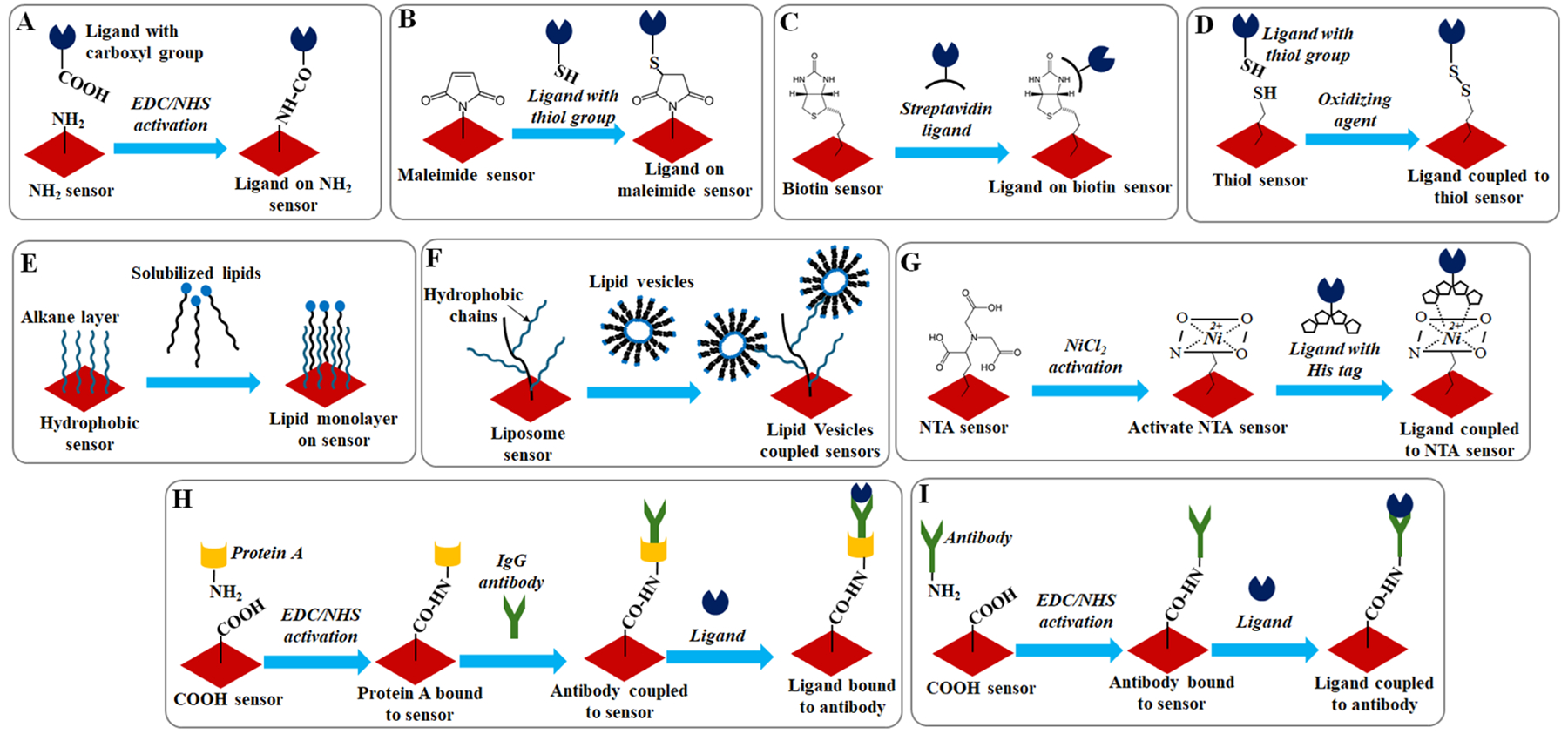
Common SPR conjugation strategies illustrating various SPR conjugation strategies used to immobilize ligands onto the sensor surface. Panel 2A shows an NH2 sensor coupled to a carboxy-functionalized ligand using EDC/NHS chemistry, a common method for covalent bonding. Panel 2B demonstrates the use of a maleimide sensor with a thiol ligand, where the maleimide group covalently binds to the thiol group of the ligand. In Panel 2C, a biotin sensor is conjugated to a streptavidin ligand, exploiting the strong biotin-streptavidin affinity for stable immobilization. Panel 2D presents a thiol sensor coupled with a thiolated ligand, forming a covalent bond between the thiol group on the ligand and the sensor surface. Panel 2E depicts hydrophobic sensors used to immobilize hydrophobic ligands through hydrophobic interactions. Panel 2F illustrates the use of liposome sensors to immobilize vesicles, a strategy particularly useful in membrane protein studies. In Panel 2G, an NTA sensor is activated with Ni ions, which subsequently bind His-tagged proteins via affinity interactions. Panel 2H shows a carboxy sensor functionalized with aminofunctionalized protein A, which then binds IgG antibodies with high affinity, allowing the attachment of specific ligands. Finally, Panel 2I illustrates a carboxy sensor functionalized with amine-functionalized antibodies using EDC/NHS chemistry, enabling the attachment of ligands with high affinity.

The successful immobilization of ligands onto the SPR sensor surface is crucial for accurate measurements. Various conjugation strategies have been developed to achieve stable and efficient immobilization, depending on the nature of the sensor surface and the ligand. These strategies typically involve the use of different chemical reactions or physical interactions to tether biomolecules to the sensor surface. The most common approaches include covalent bonding, affinity-based interactions, and hydrophobic interactions, each of which offers specific advantages for particular applications. Fig. 2 outlines several of these SPR conjugation strategies, illustrating their versatility in enabling a range of biomolecular interactions.

In the context of SPR biosensing of nucleic acids, probes typically consist of short DNA or RNA sequences of 10–50 nucleotides designed to specifically bind with a target molecule. The successful design of these probes is essential, as the stability and efficiency of the hybridization process largely depend on the probe’s sequence and structural properties. Over the years, a variety of commercial tools and guidelines have been developed to optimize probe-target binding and to prevent self-complementary regions that might interfere with hybridization^41, 42^. To anchor the probe to the sensor surface, a functional group is attached to one end of the oligonucleotide, and the spacing between the sensor surface and the recognition site is carefully controlled using spacer molecules like mercaptohexyl, mercaptoundecyl, or triethyleneglycol. Using longer alkyl chains can enhance the sensor’s sensitivity by improving the probe’s accessibility and reducing the detection limit^43^. In some cases, a sequence of non-reactive nucleotides may be added to further enhance the probe’s interaction with the target^44, 45^. Additionally, thymidine is often chosen over adenine for its weaker binding to gold surfaces, which helps maintain probe stability and improve the overall performance of the biosensor^46^. Alternative probes to conventional DNA and RNA have been explored to enhance the target recognition and sensitivity of SPR biosensors for nucleic acid detection. Among these alternatives are synthetic DNA analogues like peptide nucleic acids (PNAs)^47, 48^, PNA derivatives^49^, and locked nucleic acids (LNAs)^50, 51^. PNAs, in particular, have gained attention due to their ability to retain sequence-specific hybridization properties while adhering to the Watson-Crick base-pairing rules. PNAs also demonstrate superior selectivity compared to DNA probes, especially when detecting single-base mismatches^52^. The enhanced affinity of PNA probes for nucleic acid targets can be attributed to their reduced electrostatic repulsion, which results from replacing the negatively charged DNA backbone with a neutral pseudopeptide structure. This characteristic makes PNAs particularly useful for detecting nucleic acids with complex secondary structures, such as rRNA^53^. However, the widespread use of PNAs as biosensor probes is currently limited by the high cost of custom-synthesized PNA oligomers. The immobilization of probes onto the surface of a sensor is a critical step in the development of NA type biosensors^54,55^. This process addresses two main concerns: 1) ensuring robust and reproducible attachment of probes in accessible and well-oriented positions, and 2) minimizing background interference. Several widely used methods for probe immobilization include thiol linker adsorption, amine-carboxyl interaction, avidin-biotin interactions, hydrophobic attractions, metal ion affinity. These techniques enable the attachment of probes via a single endpoint, providing control over grafting density, which influences hybridization efficiency. One common method involves the chemisorption of probes modified with thiol groups, taking advantage of the strong affinity of thiol atoms for metal surfaces^56^. Thiolated probes can be stabilized or their density controlled by blocking agents such as 6-mercapto-1-hexanol or longer alkanethiols, which also help to reduce non-specific adsorption and improve probe orientation. The grafting density achieved with thiolated probes is high, reaching up to 4 × 10^13^ molecules/cm^2^, and can be adjusted by varying parameters including probe length, pH, and ionic strength of the immobilization buffer^43, 57–59^. Alternatively, SPR sensor surfaces can be functionalized with a layer carrying reactive groups, such as maleimide, amine, or carboxyl groups, for subsequent covalent immobilization of probes^60–64^. While functionalized surfaces in a three-dimensional matrix, such as carboxymethylated dextran, allow for a higher probe density, they can reduce hybridization efficiency due to lower probe accessibility and slower diffusion of the target molecule^65^.

Another popular approach involves using avidin or streptavidin, which binds to biotinylated probes, facilitating efficient and accessible probe immobilization^66, 67^. The streptavidin-biotin method typically results in lower probe surface density compared to thiol-based methods, but it improves hybridization efficiency due to better accessibility of the probes. However, this method can suffer from instability over repeated detection and regeneration cycles, as the anchoring protein may degrade over time.

In pursuit of further enhancing probe accessibility, alternative strategies have been proposed. For instance, poly(amidoamine) dendrimers have been used as three-dimensional linkers for oligonucleotides, allowing for higher binding capacity^68, 69^. This method is particularly beneficial for microarrays requiring long hybridization times but may offer limited improvements in real-time SPR biosensors aimed at rapid detection. Other approaches, such as the immobilization of amino-terminated probes on poly(L-glutamic acid)-modified SAMs, offer slightly lower surface concentrations but could provide a viable alternative to traditional methods, although further research is needed to assess their full potential^70–72^.

## SPR SENSORS FOR THE INVESTIGATION OF NUCLEIC ACIDS INTERACTION WITH VARIOUS BIOMOLECULES

### Nucleic Acid – Nucleic Acid interaction

**Fig. 3.**
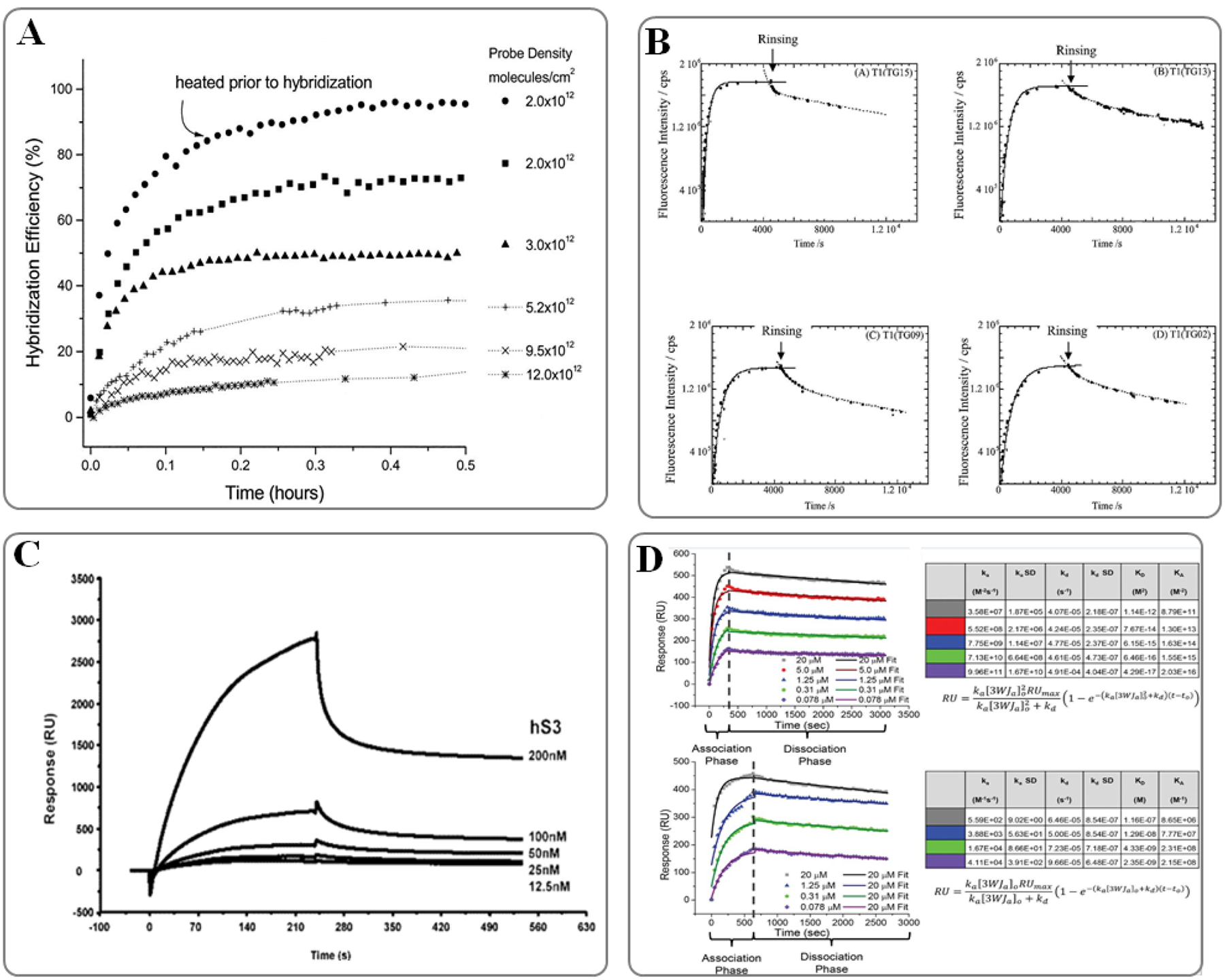
Examples of SPR sensors used for the investigation of nucleic acids interaction. A) Target hybridization kinetics as a function of probe density were analyzed, with probe density, measured by SPR, ranging from 2 × 10^12^ to 12 × 10^12^ molecules/cm^2^; figure adapted with permission from Ref #^76^ Copyright © 2001 Oxford University Press. B) Hybridization and dissociation data obtained using SPFS (represented by circles) are fitted with curves derived from the extended Langmuir adsorption model. Figure adapted from Ref #^88^ Copyright © 2001 Oxford University Press. C) Analysis of ribosomal protein S3 binding to human 8-oxoguanine DNA N-glycosylase 1; figure adapted from Ref #^107^ Copyright © 2004, American Chemical Society. D) SPR analysis of the pRNA-3WJ. Figure adapted from Ref #99; copyright © 2024, published by Cold Spring Harbor Laboratory Press for the RNA Society.

The process of nucleic acid hybridization on solid supports is inherently complex and differs significantly from that observed in solution-based systems. One key observation is that the affinity constants for NA hybridization measured using SPR biosensors often differ by several orders of magnitude from those derived using equilibrium methods^73–75^. This discrepancy arises from the limitations of the model used to describe these interactions, which was initially derived for infinitely diluted species in solution. In solid-phase interactions, the model assumes that the intermolecular distance between immobilized biomolecules is large enough to prevent direct physical interactions between them. However, in practice, the surface density of probes on SPR chips often exceeds 10^13^ molecules/cm^2^, which can lead to a range of factors influencing NA hybridization, including the surface probe density (PD), ionic strength of the hybridization buffer (I), and the specific sequence locations on the probe (Fig. 3A), as well as the probe length itself^76,77^.

The electrostatic repulsion between the negatively charged phosphate backbones of the NA probes and targets plays a significant role in the hybridization process. Cations in the buffer neutralize the negative charge on the phosphate groups, which reduces the electrostatic repulsion between the nucleic acids on the SPR surface. If the ionic strength (I) is too low for a given probe density, hybridization will not occur. On the other hand, when the probe density is low and the ionic strength is high (e.g., above 0.33 M NaCl), “pseudo-Langmuir” behavior is observed, where the hybridization efficiency becomes independent of probe density^78–80^. Between these two extremes, “suppressed hybridization” occurs, with the hybridization efficiency decreasing as the probe density increases. The “pseudo-Langmuir” regime, where the hybridization is independent of probe density, is optimal for kinetic measurements, as it reflects the behavior most similar to solution-based hybridization. This condition is also optimal for NA detection, as hybridization occurs at the highest rate under these settings. However, even in the pseudo-Langmuir regime, experimentally calculated hybridization rates and affinity constants are several orders of magnitude lower than those observed in solution^81–83^, suggesting that the hybridization process on solid supports is far more complex than the simple 1:1 interaction model can predict. Several modifications to the Langmuir model have been proposed to account for additional factors, such as the dispersion of probes, targets, or competitive interactions^84,85^.

SPR biosensors are valuable for investigating the stability of nucleic acid complexes, and they are especially effective for relative comparisons of complex stability in the context of natural nucleic acids^86^. SPR has also proven useful in studying how variations in hybridization kinetics of NAs are influenced by mismatches (Fig. 3B), both in terms of type and position^87–89^. Additionally, the interactions between chemically modified nucleic acids and their natural counterparts have been explored using SPR, helping to identify potential targets for antisense drugs and the development of novel probes for NA biosensing applications^49, 90–92^. To aid in the design of more efficient probes for NA biosensing, researchers have also studied the impact of secondary structures on DNA hybridization kinetics. It was found that the presence of three intramolecular base pairs significantly slows down the rate of DNA hybridization^93^. The findings from this work suggest that the traditional two-state model is inadequate to describe the hybridization behavior of strands with four or more intramolecular base pairs.

Further research has explored the role of cations and RNA modifications on the stability of kissing complexes using SPR. Di Primo et al. examined the interaction between RNA I and RNA II, which regulates bacterial replication, and found that the subsequent binding of the Rop protein to the kissing complex follows a 1:1 interaction model^93–95^. The formation of higher-order nucleic acid structures, such as triplexes^96^ and quadruplexes^97^, has also been extensively studied using SPR biosensors. In one innovative approach, Zhao et al. investigated the effect of fold-inducing cations on the formation rate of G-quadruplex structures^98^. They immobilized an unfolded oligonucleotide on a sensor chip and introduced a solution containing both the fold-inducing cation and a complementary oligonucleotide. The rate of G-quadruplex formation was inferred from the hybridization rate of the complementary oligonucleotide, which trapped any unfolded G-rich oligonucleotides immobilized on the chip.

Binzel et al. investigated the three-way junction (3WJ) structure in the packing RNA (pRNA) of the phi29 dsDNA packaging motor (Fig. 3D), which is essential for the motor’s thermostability^99^. The 3WJ is composed of three strands: 3WJa, 3WJb, and 3WJc. To study the interactions between these strands, the 3WJa strand was immobilized onto a neutravidin-labeled sensor surface via a biotin label. The other two strands, 3WJb and 3WJc, were introduced as analytes. SPR was used to monitor the association of the strands into the 3WJ complex and to measure the association rate constant (ka) and dissociation rate constant (kd) by observing the dissociation of 3WJ into its constituent strands. A single transition state was observed from association to dissociation across varying concentrations. To further confirm the interactions, additional SPR tests were conducted with dimers, including 3WJab, 3WJac, and 3WJbc. The results revealed that the 3WJbc dimer exhibited the highest ka, but also had the lowest kd, indicating it was the weakest and fastest-forming dimer. In contrast, the 3WJab dimer had the highest kd, suggesting greater stability. The findings concluded that the stability of the pRNA-3WJ complex is driven by the 3WJab dimer, which forms first and is stabilized by the addition of 3WJa. This study highlights the utility of SPR in elucidating the binding kinetics and stability of complex nucleic acid interactions.

### Aptamer - Target interaction

SPR is also a valuable tool for studying the binding interactions of aptamers^100^. Aptamers are synthetic single-stranded DNA or RNA molecules capable of selectively binding to specific targets, including proteins, peptides, toxins, and small molecules^101^. When employed in SPR experiments, aptamers enhance both the specificity and sensitivity of the binding analysis. One important application of SPR is in confirming the binding between an aptamer and its target molecule, a crucial step in therapeutic development. Aptamers, synthetic single-stranded DNA or RNA molecules, selectively bind to targets such as proteins, peptides, toxins, and small molecules, making them ideal for SPR applications. One key use of SPR is to confirm the binding between aptamers and their target molecules, an essential step in therapeutic development. For instance, Huang et al. utilized SPR to confirm the binding of a p53 aptamer RNA to the wild-type p53 protein^102^. The aptamer was immobilized on a streptavidin-coated sensor chip, and the binding interaction with the p53 protein was successfully observed, demonstrating the robustness of the aptamer’s binding. This confirmation of binding allowed investigators to further investigate the functionality of the aptazyme, a sensor for wild-type p53 protein, advancing its potential therapeutic applications. Valsangkar et al. employed SPR to investigate how chemical modifications of the thrombin-binding aptamer (TBA), a nucleic acid-based anticoagulant, affected its binding affinity to thrombin^103^. Using OpenSPR (Nicoya) and covalent coupling, thrombin was immobilized on a sensor surface, and varying concentrations of chemically modified TBA were introduced. The binding interactions were analyzed using a 1:1 binding model with TraceDrawer software. The results showed negligible changes in the dissociation constants across the different TBA modifications, indicating that the chemical alterations did not significantly affect the TBA-thrombin binding.

Aptamer binding studies can also be conducted without directly immobilizing RNA on a sensor surface. Li et al. explored RNA microarrays where the aptamer of interest was ligated to single-stranded DNA (ssDNA), which was immobilized onto a chemically modified gold surface (104). The RNA aptamer was then ligated to the ssDNA using T4 RNA ligase. To minimize non-specific binding, excess reagents were removed by rinsing with RNase H, which regenerates the ssDNA backbone, allowing the ligation of a new RNA aptamer without the need to immobilize fresh ssDNA. This process highlights an efficient method for reusing sensor surfaces in aptamer-binding studies, offering flexibility in microarray applications. The surface ligation methodology eliminates the need for modified ssRNA and background proteins such as streptavidin, offering a more streamlined approach for aptamer studies. It also enables the simultaneous observation of multiple aptamers. Authors demonstrated the effectiveness of this approach by developing a five-component RNA microarray targeting potential aptamers for protein factor IXa (fIXa).

### Nucleic Acid-Protein interactions

SPR sensors are frequently employed to study interactions between DNA-binding proteins^105^, particularly those involved in critical cellular processes such as DNA replication and repair^106, 107^ as well as transcription^108, 109^. Notably, the interaction parameters derived from SPR analyses on solid supports align closely with results obtained using solution-based techniques, demonstrating the reliability of SPR for investigating these fundamental biological processes. Also, SPR biosensors have been utilized to study RNA-binding proteins involved in various biological functions (Fig. 3C), such as translation regulation^110^, binding to mRNA and tRNA^111,112^, interactions with ribosomal RNA (rRNA)^113^ and translation initiation factors that interact with the 5’-cap of mRNA^114^. The application of SPR imaging has been pivotal in studying transcription factor (TF) binding, particularly for multiplexed analyses of TF interactions with various gene sequences^115^. These studies commonly utilize short dsDNA arrays or entire gene promoters^116, 117^. Although steric hindrances on crowded surfaces can influence the kinetic parameters in promoter-level analyses, such approaches provide rapid screening capabilities to identify TF binding sites across whole genes.

**Fig. 4.**
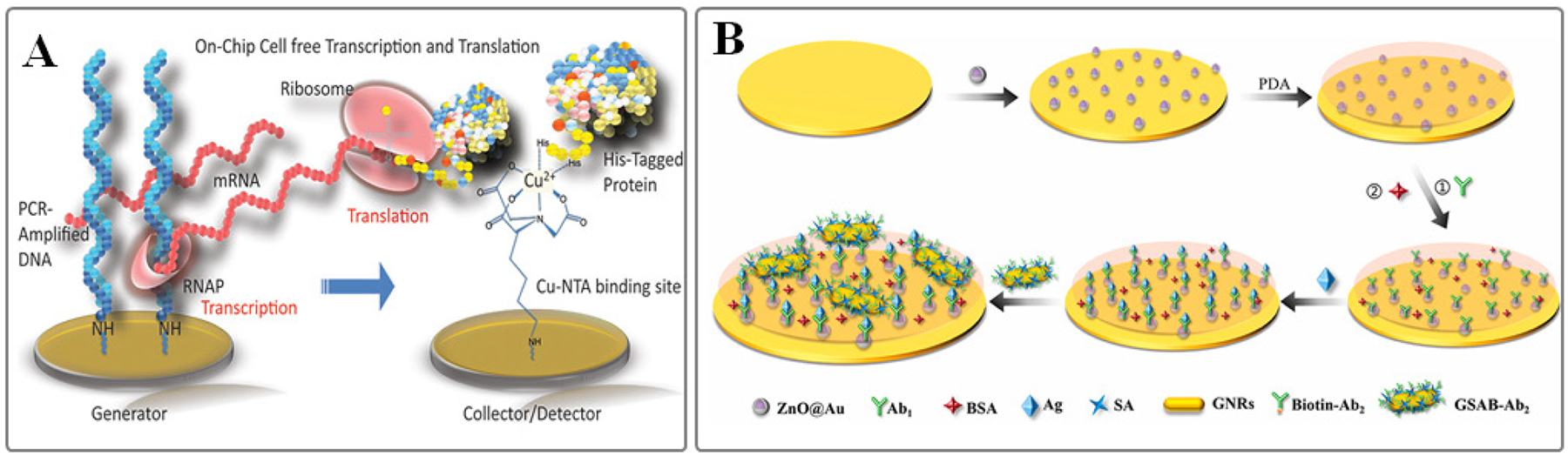
**A**. Schematic illustration of protein microarray synthesis from a DNA microarray via surface transcription and translation. Herein, dsDNA templates encode His6-tagged proteins captured by Cu(II)-NTA on detector elements. Figure adapted from Ref #^124^ Copyright © 2004, American Chemical Society. **B**. Example of sensitive SPR biosensor using ZnO@Au nanomaterial and a classical sandwich strategy with biotin-streptavidin for secondary signal amplification to detect human IgG (hIgG); figure adapted with permission from Ref #^67^ Copyright © 2022 Elsevier B.V.

SPR biosensors also excel in unraveling complex multiprotein-DNA interactions. For instance, Neo et al. utilized this technology to investigate the cooperative binding of two transcription factors, ER and SP1, to a DNA oligomer with distinct segments^118^. Their study, conducted in nuclear extracts with physiological concentrations of ER and SP1, confirmed the formation of a ternary complex involving ER, SP1, and the composite DNA, showcasing the power of SPR biosensors in studying intricate molecular interactions. Modern SPR biosensors offer the ability to detect minute changes induced by enzymatic processes, such as the elongation or cleavage of NAs immobilized on the sensor surface (Fig. 4). However, distinguishing the enzymatic action’s kinetics from the coincident association or dissociation of the enzyme itself remains challenging. Consequently, most studies focus on binding assays, which assess the affinity of inactivated proteins for substrates with varying sequences or structures^119^.

By employing different analyte injection approaches, researchers can gain a deeper understanding of molecular binding capacities and affinities. In a study by Fang Teh et al.^77^, four SPR assay formats (direct, competition, dissociation, and sandwich) were applied to analyze the binding interactions between estrogen receptors (ER, α and β subtypes) and estrogen response element (ERE) sequences. Each assay was conducted on a biotin-coated sensor surface with streptavidin bound, enabling the immobilization of ERE sequences. In the direct assay, ERE sequences were immobilized on the sensor surface while ER was injected as the analyte, allowing quantification of ER binding capacity to the ERE sequences. For the competition assay, ERE sequences were similarly immobilized, but ER was injected alongside a competing ERE sequence. This approach enabled the determination of ER affinity across different ERE variants, by observing competitive binding effects. In the dissociation assay, after initial injection of ER as in the direct assay, a buffer was introduced to induce ER dissociation from the ERE sequences, providing data that could be used to calculate binding stoichiometry. Lastly, the sandwich assay introduced an additional binding partner, anti-ER, following ERE and ER injections. This method allowed examination of the protein-DNA complex’s influence on the ER’s binding ability to an additional partner, revealing the complex’s effect on further interactions. Each assay type thereby highlights unique facets of ER-ERE interactions, underscoring SPR’s adaptability for comprehensive binding analyses.

Both cleavage and elongation of immobilized NAs can be monitored indirectly. For instance, when enzymatic action generates single-stranded nucleic acids, a complementary strand^120, 121^ may be introduced for semi-quantitative measurement of the enzyme’s activity. Vaisocherova et al. demonstrated an indirect approach to monitor the integration of viral dsDNA into the host genome by HIV integrase^122^. In their method, duplexes were immobilized on the sensor surface to represent the host genome, and after washing steps to remove both the enzyme and unbound viral dsDNA, the amount of integrated dsDNA was measured.

Jorgensen et al. studied the activity of human poly(ADP-ribose) polymerase-1 (PARP-1) on oligodeoxynucleotide substrates mimicking various double-stranded DNA structures^123^. Their results indicated that PARP-1 preferred the duplex structure over hairpin and loop forms, which correlated with a higher amount of incorporated ADP per enzyme molecule.

**Fig. 5.**
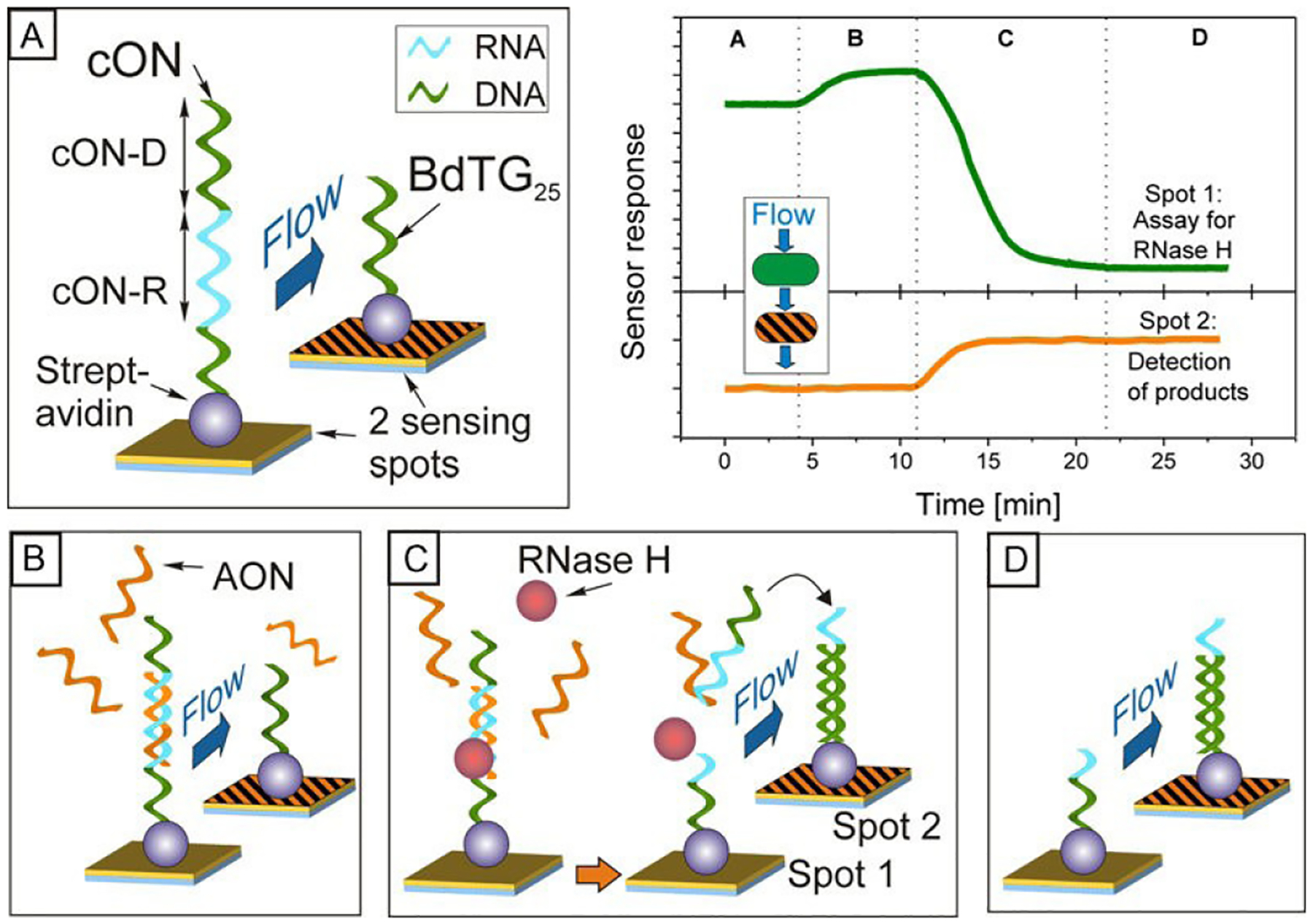
Overview of the dual SPR-based assay for studying RNase H activity. (A) Initial baseline in buffer, where no interactions occur with immobilized oligonucleotides. (B) Injection of tested antisense oligonucleotides (AONs). The formation of the cON-R:AON duplex is detected in the upstream area, with no response in the downstream area. (C) RNase H mixed with AONs is injected. A reduction in the upstream sensor response indicates AON-mediated RNase H cleavage of cON-R, while cleaved fragments are recaptured in the downstream area, causing an increase in its sensor response. (D) Final baseline in the buffer. The change between the initial and final baselines in both sensing areas (a decrease upstream and an increase downstream) reflects the amount of cleaved probes. Figure adapted with permission from Ref #^125^ Copyright © 2010 Elsevier B.V.

Only a few studies have successfully monitored enzymatic action in real-time. Corn’s group demonstrated that catalytic and kinetic constants could be derived from the enzymatic cleavage kinetics measured by SPR biosensors^124^. They applied a simple model that combined biomolecular adsorption and surface enzyme kinetics, characterized by three rate constants (ka, kd, and kcat), along with a diffusion parameter. Similar model was used to study RNA strand cleavage in RNA/DNA hybrid duplexes by RNase H (Fig. 5). Sípová et al. also investigated RNase H activity and proposed using an additional sensing channel downstream to detect cleavage products, offering further insight into the cleavage reaction by simultaneously measuring product concentrations^125^.

In the recent works, Yang et al. investigated the binding affinity of the nucleocapsid protein from the severe acute respiratory syndrome coronavirus (SARS-CoV), which exhibits a strong binding affinity for the leader sequence of the SARS-CoV genome, through DNA-RNA interactions^67^. In their study, a biotin-coated sensor was employed to immobilize streptavidin, which subsequently facilitated the binding of biotinylated DNA. The 3′-end of the RNA, complementary to the immobilized DNA, was hybridized at varying temperatures, leaving the 5′-end of the RNA exposed to interact with the nucleocapsid protein. While it is common to immobilize the RNA directly onto the sensor chip, this hybridization approach, where RNA is attached to DNA, offers the added advantage of enhanced stability provided by the DNA, improving the reliability of the binding analysis.

### Nucleic Acids - Small Molecules interactions

Nucleic acid-small molecule interactions are of significant interest, particularly for the development of drugs that act as minor groove binders or intercalators. Although SPR biosensors offer an attractive tool for studying these interactions, the low molecular weight of the binding molecules poses a challenge for direct monitoring. To address this issue, immobilization techniques using a dextran layer have been implemented^126^, which increases the available surface area for binding, thus enhancing the sensor response. Additionally, increasing the number of binding sites by repeating the sequence of interest multiple times in the probe can also improve sensitivity^127^. However, this approach may introduce variability in observed affinity due to differing accessibility of binding sites along the probe^128^.

SPR biosensors have been employed to investigate NA interactions with a variety of compounds associated with diseases such as cancer, inflammation, thrombosis, osteoporosis, and viral infections^129–133^. Furthermore, SPR has been utilized to measure differences in small molecule affinity towards various NA sequences^134,135^. A growing area of interest is the interaction of small molecules with G-quadruplex DNA structures^136^, including porphyrins^131^ and new classes of G-quadruplex binding ligands^137^. These interactions are particularly relevant in the context of cancer therapeutics, as certain small molecules targeting G-quadruplexes may function as telomerase inhibitors, providing potential therapeutic avenues^138,139^.

In addition to immobilization techniques, the incorporation of nanoparticles into SPR sensors can significantly enhance sensitivity of the SPR biosensors. Kazmi et al. employed doxycycline-coated gold nanoparticles (doxy-AuNPs) to detect doxycycline levels^140^. The presence of free doxycycline enhanced the binding of doxy-AuNPs, which in turn amplified the SPR response. As the concentration of doxycycline increased, the doxy-AuNPs aggregated more rapidly, resulting in a stronger SPR signal and improved detection sensitivity.

## CONCLUSION AND PERSPECTIVES

SPR has emerged as a powerful and versatile tool in nucleic acid nanotechnology, providing invaluable insights into the kinetics, thermodynamics, and specificity of nucleic acid interactions. Its ability to offer real-time, label-free analysis makes it ideal for studying complex systems, such as DNA and RNA nanostructures, aptamer-based sensors, and nucleic acid-protein interactions. SPR has facilitated advancements in the design and optimization of nanostructures for targeted drug delivery, diagnostics, and biosensing by enabling precise characterization of binding events and molecular interactions. Furthermore, recent innovations, such as dual-SPR assays and integration with nanoparticles, have expanded its utility in probing enzymatic activities and enhancing detection sensitivity. As nucleic acid nanotechnology continues to evolve, SPR will undoubtedly remain a critical analytical tool, driving innovations in fields ranging from therapeutics to synthetic biology.

SPR biosensors have been widely employed to study various Nucleic Acid interactions, including NA–NA, NA–Protein, and NA–Small molecule binding, advancing our understanding of complex biological processes and facilitating the development of new therapeutics. One of the main advantages of SPR biosensors is their ability to measure binding events in real-time without the need for labeling the interacting molecules, which is a significant benefit for studying molecular interactions in their native state. As a result, SPR biosensors have been routinely used to quantify the kinetic parameters of molecular interactions with considerable amount of work has been focused on improving the sensitivity and sequence specificity of NA SPR sensors, enabling them to detect nucleic acids at attomolar concentrations and distinguish single nucleotide differences in targets. Despite the large number of studies in controlled buffer conditions, there are fewer reports documenting the performance of NA SPR sensors in complex biological samples or comparing their detection capabilities in both buffer and real-world environments.

Moreover, the quantitative comparison of results across different studies is challenging due to the influence of multiple factors, such as the sensor platform, probe design, functionalization methods, detection techniques, and the complexity of the sample matrix. Multiple published reports suggest that one of the main factors limiting the performance of NA SPR sensors in complex samples is the non-specific adsorption of molecules, which contributes to background noise and interferes with accurate measurements. Therefore, while progress has been made, there is still a need for further development of NA SPR sensors to overcome these challenges and enable their routine use as reliable bioanalytical tools in complex biological environments.
